# Transgender population’s experiences with regard to accessing reproductive health care in Kwazulu-Natal, South Africa: A qualitative study

**DOI:** 10.4102/phcfm.v11i1.1933

**Published:** 2019-07-10

**Authors:** Zamasomi P. B. Luvuno, Busisiwe Ncama, Gugu Mchunu

**Affiliations:** 1 Centre for Rural Health, School of Nursing and Public Health, University of KwaZulu-Natal, Durban, South Africa; 2 School of Health Sciences, University of KwaZulu-Natal, Durban, South Africa; 3 Discipline of Nursing, School of Nursing and Public Health, University of KwaZulu-Natal, Durban, South Africa

**Keywords:** transgender population, transgender health, transgender, HIV, transphobia, health care access, health care accessibility

## Abstract

**Background:**

The transgender population has unique health risks, including increased risk of mental illness, substance abuse, suicide and a high prevalence of human immunodeficiency virus (HIV). Worldwide studies indicate that this population is marginalised and faces barriers in accessing health care. In South Africa, there is limited information and research on the transgender population’s interaction with health services.

**Aim:**

The purpose of this study was to examine the experiences of the transgender population in accessing health care facilities for sexual and reproductive needs.

**Setting:**

The study took place in KwaZulu-Natal province of South Africa.

**Methods:**

A qualitative study combining phenomenological and critical ethnographic approaches was conducted to explore the experiences of the transgender population in the health care setting. Critical ethnography was chosen because it is an emancipatory method that highlights the plight of disenfranchised groups, and phenomenology was used to illuminate experiences of the transgender population. Purposive snowball sampling was applied to select nine transgender participants who had experiences of contact with a health care setting. Data collection was performed through semi-structured interviews and a focus group discussion.

**Results:**

Participants provided details about the paucity of facilities, resources and targeted programmes to cater for the transgender populations’ sexual and reproductive health needs. The participants engage in high-risk behaviour, comprising unprotected sex and use of cross-gender hormones without medical supervision. Furthermore, the participants reported experiences of hostile and discriminatory behaviour by healthcare workers.

**Conclusion:**

It emerged that there is a paucity of resources and knowledge to provide appropriate health care services to the transgender population, resulting in adverse experiences. Policies on transgender care and training of health workers will contribute towards improvement of health care access for the transgender population.

## Introduction

*Transgender* people are individuals whose current gender identity differs from the birth assigned sex and expression.^1^ The term ‘transgender female’ refers to transgender people assigned male sex at birth who are on the transgender spectrum identifying as women, while ‘transgender male’ describes transgender people assigned female sex at birth who are on the transgender spectrum identifying as men.^1,2^

The transgender population is one of the most marginalised social groups globally, and is often targeted for mistreatment and discrimination. This occurs even in societies with progressive human rights policies.^3,4,5^ The mistreatment and discrimination experienced by the transgender population is referred to as *transphobia*, which is described as an irrational fear and hatred of transgender persons because they do not conform to societal gender norms.^6,7^ This can result in the exclusion of the transgender population by not assigning them a legal identity, resulting in challenges such as lack of employment or social support and limited access to quality health care.^4,8,9^ Studies have indicated that the transgender population faces a number of structural and systemic barriers to accessing quality health services.^9,10^ Structural barriers they encounter include failure of the health care system to provide a conducive environment, such as unisex bathrooms and appropriate arrangements for inpatient transgender patients.^11^ Systemic barriers include erasure through failure to acknowledge the existence of the transgender population as patients within the health system.^12^ The erasure can be passive, through lack of knowledge, data, policies and practice guidelines relating to the transgender population, resulting in a paucity of programmes that cater for transgender patients,^12,13^ and there can also be active exclusion in the form of hostility and verbal abuse intended to cause discomfort and harm to transgender patients, thereby alienating the transgender population and, eventually, resulting in their avoidance of health care facilities.^9,12,13^

Yet, the transgender population has unique health risks, such as increased risk of mental illness, substance abuse, suicide and a disproportionately high prevalence of the human immunodeficiency virus (HIV). Notably, the transgender population has a disproportionately high HIV acquisition rate even in areas with low HIV prevalence.^4,14,15,16^ It is reported that the risk of contracting HIV among the transgender population is fourfold that of the general population.^11,17^ A meta-analysis conducted in the United States across 10 states indicated a 27% HIV sero-prevalence rate in the transgender female population, whereas the general prevalence in the population was only 0.6%.^18,19^ Furthermore, transgender individuals rely on hormones and surgical procedures to reconcile their anatomy and physiology with their gender identity, and this requires medical access.^13,20^ Because of the paucity of targeted programmes and the discrimination experienced in health care settings, the transgender population opts to avoid health care facilities and instead obtain cross-gender hormones without medical supervision. Prevalence of unsupervised hormone use in transgender populations ranges from 29% to 63% and is associated with health risks such as hypercoagulability and thromboembolism, and decreased insulin sensitivity.^20,21^ It is also reported that the transgender population often resorts to self-performed surgery, including orchiectomy among transgender females and mastectomy among transgender males.^21,22^

It is possible that the African transgender population faces challenges similar to those encountered by corresponding populations in middle-income countries, and is exacerbated by criminalisation of lesbian, gay, bisexual and transgender (LGBT) communities in most African countries.^23^ The transgender population in the Southern African region is highly vulnerable to HIV infection, in view of the high prevalence rates in the general population.^19,24^ Although little research has been conducted on the South African transgender population, the scholastic literature that exists confirms that the transgender population is marginalised and faces barriers in accessing healthcare services, mainly because of the lack of policies or programmes and limited skills among health care workers in dealing with sexual health.^25,26,27^ A study by Stevens^28^ on transgender patients in the South African setting indicated that the patients were mistreated in health care facilities, with 60% of the participants reporting poor reception, provider insensitivity and hostility.

### Problem statement

During the apartheid period (1948–1994) in South Africa, there were a number of statutes that criminalised and denied rights to sexual and gender minority populations.^29^ The LGBT populations were thus left without rights or legal protection of any kind. Adoption of the new South African Constitution in 1996 and the Bill of Rights, section 27(1), established, for the first time, protected and guaranteed rights for all, including the LGBT population. Among these rights was access to health care services for all South Africans, with the stipulation that no person shall be refused treatment or provided with inferior care.^30^ Despite the legal rights, evidence suggests that the LGBT population encounters numerous structural and systemic barriers that hinder their access to quality health care services.^28,31^

### Purpose of the study

This study describes the experiences of transgender individuals and their responses regarding accessing health care facilities for sexual and reproductive needs. The discussion is presented in an endeavour to better inform health care workers about the needs of the transgender population.

### Definition of key concepts

*Transgender* is an umbrella term that is widely used to refer to a diverse group of individuals whose gender identity and expression differ from culturally defined categories of gender, or to denote gender that does not conform to societal gender norms.^1,32^

*Cisgender* (often abbreviated as *cis*) is a term for people who have a gender identity that matches the sex that they were assigned at birth.^5^

*Transphobia* is defined as societal dislike and discomfort with people whose gender identity and/or gender expression do not conform to traditional or stereotypic gender roles.^33^

*Gender affirmation* is a process of affirming one’s gender identity by social expression, psychological validation, medically (hormones, surgery, etc.) and/or legally (legal gender and name change).^34^

## Research design and methods

Critical ethnography starts with an ethical desire to address injustice and lack of fairness in a particular lived domain, driven by principles of compassion and desire to contribute to the alleviation of human suffering.^35,36,37^ In South Africa, the transgender population is a marginalised group, despite protective national legislation.^26,38^ In this study, a critical ethnographic approach was deemed appropriate in seeking to give a voice to the transgender population, challenging the status quo, highlighting their plight and outlining their health care experience in relation to their everyday lives.^35,39^ Phenomenological orientation was used to illuminate the experiences of transgender patients in navigating the health care system. Phenomenology is the most appropriate qualitative method for this purpose because it focuses on seeking to understand the essence and meaning of individuals’ lived experiences of a phenomenon so as to reveal distinctive meanings – in this case, experiences that pertain to accessing sexual and reproductive health care in health care facilities.^35,40^ Following social constructivist paradigms, the researchers sought to build a collaborative relationship with participants, acknowledging and bracketing researcher biases, and recognising transgender identity as a central organising concept in the participants’ lives.^37^ According to the constructivist perspective, knowledge is socially and culturally constructed, and reality cannot be discovered because it does not exist until it is created in relation to a given experience.^41^ Realities vary, are multiple and complex, and the researcher thus relies on the utterances of the participants on how to view the situation, as the meanings are determined by history and the societal milieu. All research biases were documented using *memoing* – writing ongoing notes exploring how researcher biases were shaping the interpretation of data.^40,42^

### The study population and sample

Initially, 16 participants were recruited, of which nine met the inclusion criteria that were as follows: age 18 years or older, self-identified as transgender and having had some contact with health care services. Eight of the participants had completed high school (matriculation), and one was in her matriculation year at the time of data collection. Of the eight participants who had competed matric, six were pursuing vocational studies, one was employed part-time and one was unemployed; none had any private medical insurance. Participants earned below R2000.00 ($143.00) per month. Eight participants identified as transgender females and one as transgender male. Seven of the eight participants who were transgender females said they were heterosexual (attracted to men), and two said they were bisexual (attracted to both men and women). All participants were black South Africans and were purposefully selected as being likely to seek health care services in government health care facilities. Four participants were from rural areas, three from an urban setting and two from peri-urban settings (see Table 1).

**TABLE 1:** Demographics of the participants.

Pseudonym	Age (years)	Sex assigned at birth	Gender identity	Sexual attraction	Race	Employment	Education	Residential Setting
Mancane	19	Male	Female	Attracted to men	African	Scholar	Grade 11	Peri-urban
Nomfundo	26	Male	Female	Attracted to men	African	Unemployed	Matric	Urban
Zibuyile	21	Male	Female	Attracted to men	African	Student	Matric	Rural
Nobuhle	23	Male	Female	Attracted to men	African	Student	Matric	Rural
Pretty	25	Male	Female	Attracted to men	African	Employed (part-time)	Matric	Peri-urban
Sibusiswe†	19	Male	Female	Attracted to men	African	Student	Matric	Rural
Mandla†	24	Female	Male	Attracted to men and women	African	Student	Matric	Urban
Philile†	23	Male	Female	Attracted to men and women	African	Student	Matric	Rural
Nomusa†	24	Male	Female	Attracted to men	African	Student	Matric	Urban

† , Focus group discussion participants.

### Sampling procedure

Snowball sampling was used to find the prospective participants and purposive sampling was then followed to select the participants who met the inclusion criteria. Snowball sampling was preferred for this study as the transgender population is a vulnerable and hidden minority group.^39^ In addition, phenomenological studies rely on purposive sampling because participants must be selected intentionally on the basis of having experienced the phenomenon under investigation.^35,37^ In this study, snowballing was adjusted slightly to preserve the confidentiality of the participants. For the initial step, the researcher requested the seed participant, who was known to the author socially before the study, to get permission from acquaintances before forwarding their names to the researcher. After a week’s delay, to give the seed participant time to contact the acquaintances, the researcher then called the seed participant to enquire if those who had been approached were willing to participate. Once this assurance was given, the researcher obtained contact details of the potential participants and invited them to take part in the study. Each participant was given R50.00 ($3.60) to cover the cost of travel to the interview venues.

### Data sources and data collection

Consent was obtained from each participant and they were provided with an information sheet explaining the purpose of the study, written in isiZulu. Data were collected on demographic particulars for each participant: age, gender, sexual orientation, geographic setting and level of education.

### Interviews

Five individuals participated in the individual semi-structured interviews, and each interview lasted between 45 minutes and 90 minutes. The following two main broad questions were posed: ‘Tell me about the time you first discovered that you may be different from other boys and girls?’ and ‘what were your experiences at the health care facilities when seeking care for sexual or reproductive health?’ Recruitment was stopped at five individuals because no new data were emerging.^43^ A single interview was conducted with each participant to gain an understanding of the participants’ interpretation of their experience at that moment in time.^40^

### Focus group discussion

Four participants took part in one focus group discussion, conducted in a venue at an academic institution. The purpose of the focus group was to further explore and triangulate the thematic findings from the individual interviews.

The individual interviews and the focus group questions explored the participants’ experiences when attending health care facilities for sexual and reproductive health care. A section of the questions requested participants to give, in isiZulu, a description of transgender identity and definitions of the LGBT population in the South African setting. The interviews and the focus group discussion were audio-taped. Once the audio tape was switched off, questions were invited from the participants. The researcher used the following question to facilitate the interview: ‘What were your experiences at the health care facilities when seeking care for sexual or reproductive health?’

### Data analysis

The audio-taped discussions were conducted in isiZulu, transcribed in isiZulu and then translated into English. The isiZulu verbatim responses were translated by an isiZulu first language speaker who had studied English as his or her first language, then was back-translated and finally confirmed by the researcher and the supervisors.

In the first step, the data were coded for reducing them to general, broad codes using the phenomenological coding process of horizontalisation of data (developing a list of non-repetitive significant statements, all with equal value).^40^ The second step involved meeting with the research team that consisted of two PhD trained researchers who supervised my PhD study, to develop meanings or themes, or textual description, which were written verbatim. The third step involved development of a structural definition of how the phenomenon was experienced. The data were then reduced to information and significant statements, which were combined to form themes. The themes were then developed to textual descriptions of what was experienced. Lastly, a structural description was used to convey the overall essence of the experiences.^40,44^ Through constant comparison,^37^ the researchers identified both common and variant themes across the interview data. The focus group discussion transcripts were coded using the themes that emerged and confirmed from the individual interviews.

### Trustworthiness

Trustworthiness was established by applying credibility, dependability, transferability and confirmability criteria described by Cresswell.^37^ *Credibility* was assured by using a purposive sample of participants who self-identified themselves as transgender and had accessed health care facilities for sexual and reproductive health needs. The audio recordings and transcripts were shared with the supervisors to confirm codes. Sufficient description of the research and the research findings were provided for them to be evaluated for applicability to settings other than KwaZulu-Natal. The themes that emerged from the individual interviews were confirmed with the focus group participants.^37^ A reflexive journal was kept by the researcher to document biases throughout the research process.^45^ *Dependability* ensured consistency and the ability of other researchers to be able to replicate the study.^46^ This was achieved through the provision of the details on study design, methods used for data analysis and data gathering techniques. *Transferability* was achieved through thick description of context and purposeful selection of the participants. Authenticity was supplemented by inclusion of verbatim quotes from research participants to describe findings, which assisted to achieve transferability.^47,48^ To ensure *conformability*, collected information was verified with the research supervisors and the study participants throughout the process of data collection.^47^

### Ethical considerations

Ethical clearance was obtained from the University of KwaZulu-Natal Biomedical Research Ethics Committee (BE 115/14).

## Results

Two main themes and five sub-themes emerged from the data analysis. The first main theme was sense of self and its sub-themes were (1) discovery and definition of self, (2) body alignment and (3) sexuality. The second main theme was navigating the cisgender health system and its sub-themes were (1) health care worker ignorance and (2) micro-aggression.

### Sense of self

Participants described experiences of a period of discovery of their gender identity, which will be highlighted in the sub-themes below.

#### Discovery and definition of self

The participants used various terms to describe their experiences of feeling they were different from other boys and girls from the age of 13 years. They described experiences of feeling that they were in a wrong body and/or wrong gender. However, in each case, there was a sense of confusion as to why they were feeling alienated towards their gender assigned at birth, without any reference point or explanation for their feelings. Almost all participants spoke of feeling ‘trapped’ in a wrong body. The confusion was compounded by teasing from peers and family pressure to conform to the societal gender norms, which are evident in the following quotes:

‘At that stage, I thought I was gay. But when I was alone, I wanted to be a girl. The girls who were with me in high school, that were my friends from primary school, started to develop. They started to have boobs, they were plaiting their hair, doing all these girly things, and I would wish that I could do what they were doing. I was very confused.’ (Nomfundo, 26 years, unemployed)

‘I’m a girl, I feel like a girl. I really want to have breasts and be a proper girl. I envy the girls, and I think, ‘why can I not have breasts and hips like them’. I hate my penis! I would cut it off if I could! I always hide it when I get dressed.’ (Pretty, 25 years, works part-time)

‘Everybody [at school] was like, ‘Moffie! Moffie!’ I then went to my granny to tell her. Instead of consoling me, she said, why are you playing with girls? That is why they call you a moffie.’ (Zibuyile, 21 years, student)

‘This is the way I am, I’m a girl, trapped in this body. I really want to change.’ (Mancane, 19 years, scholar)

There was no understanding or explanation of the feelings of being in a wrong body and gender. Some participants reported getting confirmation and acknowledgement of what they were going through, from the mass media:

‘You know, when I was 17 one day, Oprah [Winfrey] came on. She was talking to this blond lady. She [the lady] said she was a man, married. She wrote a book called ‘He is not there’, meaning that the husband, that the wife thought she was married to, is not there. This lady started to talk about transitioning. I thought this is it! They started talking about hormones and surgery.’ (Nomfundo, 26 years, unemployed)

The participants also credited the non-governmental organisation (NGO) that deals with the LGBT communities for clarifying what was happening to them, and for giving assistance and coaching on how to define themselves in acceptance of their transgender identity. One participant, Pretty, gave a definition she was given by the NGO:

‘[*B*e]cause there’s a difference in transgender, there’s a transwoman and a transman; transman is a person born with woman’s organs but who doesn’t have feelings that [are] of a woman, and then transwoman is a male born with male organs but doesn’t have man hormones. That’s why it is called transgender, and our bodies feel like they have been trapped in the wrong body. Because our feelings do not correspond, that is why we end up taking hormones, so that they help us build those body parts that we don’t have. If you are a transwoman, build those womanly parts; if you a transman, build manly organs.’ (Pretty, 25 years, works part-time)

‘Through the workshops, you end up accepting that your body is the way it is, unless if you have money to transition.’ (Zibuyile, 21 years, student)

Realising what was happening brought relief, followed by a desire to then align the body with the experienced gender. Wanting to bring about alignment between their body and their internal feelings was what drove some of the participants to seek medical advice.

#### The emergent desire for body alignment

The participants all described an overwhelming desire for alignment between outward physical appearance and internal felt gender. The misalignment caused them a great deal of distress, which, in most instances, stimulated and necessitated contact with the health care system in an effort to transition. However, because of the lack of transgender-friendly health care services, some resorted to extreme measures, such as self-performed surgery, hiding the undesired body parts, obtaining illicit hormones or/and oral medication in an effort to transition to the felt gender identity, as evidenced in the following quotes:

‘I had this overwhelming feeling in 2014 that I want to remove my penis; I didn’t want it anymore. I told my mom and she said its better I go to the doctor. I then went to a doctor in a community health centre (CHC) and the doctor said there’s nothing he can do.’ (Pretty, 25 years, works part-time)

‘It got so bad, that at 15, I got myself a razor blade. I decided I’m going to cut my private area, cut away my male organs. In my mind, I thought I will bleed so heavily, my granny will take me to the hospital, where they will cut off my private parts and make me a girl.’ (Nomfundo, 26 years, unemployed)

‘Whenever I leave the house I will always tie my penis up, I’ve told myself that obviously, no one sees that I have it but I really don’t like it, I have gotten used to it even though I don’t like it.’ (Nomusa, 24 years, student)

‘I wear two tight panties so it [penis] lies flat.’ (Nobuhle, 23 years, student)

‘I started taking them [cross-gender hormones] in 2011, but it was not prescribed by the doctor, my friend was taking them and I also started taking them. But they don’t give me what I want, I just gain weight. It was 2 mg Estopause because my friend was taking that.’ (Nobuhle, 23 years, student)

#### Discovering sexuality

Participants shared experiences of discovering sexuality and sexual attraction. It is part of the fabric in definition and realisation of the true self. There is an element of validation of gender identity, interwoven with confirmation of sexuality and sexual orientation. Seven of the nine participants identifying as transgender females indicated that they were heterosexual and attracted only to straight men, explaining that they believed themselves to be women and therefore could not engage sexually with gay men because that would mean they were homosexual. Most of those who identify as transgender females indicated to have engaged in mainly receptive anal sex and oral sex. Their bodies and sexual organs remained those of the birth gender: in this case, male. Some reported that as they had begun using hormones they were mostly unable to sustain an erection. The only participant who self-identified himself as transgender male indicated that he has sexual relationships with both men and women, and, therefore, he is likely to get pregnant. One transgender female participant said she was fluid, and was bisexual and versatile, engaging in both receptive and penetrative anal and oral sex. One participant disclosed engaging in sexual relationships because of a promise of money to fund hormones:

I can say that I’m bisexual, because in high school I had a girlfriend, one or two and after high school, I started seeing this guy and stuff, and I fell in love with him, but still had a side chick and guy there on the side, so when I’m tired of the guy, I’d go find a girl. So I’m both ways.’ (Mandla, 24 years, student)

### Experience of navigating the cisgender health care system

Cisgender is defined as having a gender identity matching the sex assigned at birth.^49^ Participants reported approaching a health worker for assistance with sexual health problems, such as sexually transmitted infections (STIs) and HIV testing, and requests for transitioning was met with confusion. In some instances when seeking care for STIs, because of fear of reprisals, the transgender identity was not disclosed, nor was the gender of the partner; they just went along with the health care workers, who assumed cisgender and heterosexual messaging during counselling and history taking:

‘I was in Grade 10, I started having a boyfriend and he was much older than me and we found ourselves being sexual, and we didn’t use protection and I contracted an STI which was warts. It was the first experience that I had to go to visit a healthcare facility. I got to the clinic, when I got there the first nurse who consulted with me didn’t understand what was happening because these warts were in the anal area, it was like a pimple and I didn’t understand either what was happening.’ (Pretty, 25 years, works part-time)

‘The only problem we have is that there is no protection for us when we do oral sex because we enjoy oral more and it’s a perfect substitute for anal because anal can be painful. Once I got a sore throat after oral sex, I went to the clinic, I did not tell them about the sex, and she (nurse) said I had strange patches on my throat. I’m sure it must have been an STI.’ (Sibusiwe, 19 years, student)

#### Health care worker ignorance

Participants reported having experienced an overwhelming desire to transition and be in alignment with their felt gender identity. But when they asked for transition and gender-affirming services, they were met with confusion from health care workers who were unable to offer care, advice or appropriate referral. Participants were sent from pillar to post and seen by numerous health care workers without success, much to their personal distress and frustration:

‘I first started trying to transition by going to government facilities. I went to the local clinic. I assumed they know how to help me. I heard from some of my friends that you can [use] contraceptives to get female hormones … so I went to the clinic. The nurse asked what is wrong with me.’ (Nomfundo, 26 years, unemployed)

‘She [nurse] then wrote me a letter and said, ‘hambo buza esibhedlela’ (go to the hospital and ask them there). On the letter, she wrote that I’m a hermaphrodite. She did not examine me, not sure why she thought I was a hermaphrodite.’ (Nobuhle, 23 years, student)

‘The doctor then said, ‘I will refer you to another hospital (names the hospital) maybe they will help you there.’ I went there. Same story. The health workers were confused. I just decided to stop. I forgot about the medical help (to transition). I decided to just live as a woman.’ (Pretty, 25 years, part-time employment)

#### Health system micro-aggression

Participants reported encounters with hostile health services, in instances of what is referred to as *micro-aggression*: distasteful treatment (whether intentional or unintentional) that communicates hostility, prejudice and insult towards members of oppressed groups, also defined as verbal, behavioural or environmental enactment of humiliation.^50^

Participants reported experiences of denial of privacy: violation of bodily privacy through health care worker voyeurism and deliberate exposure of the transgender-status patient to other patients, and being made a spectacle to other patients and health care workers. In addition, they mentioned having been treated as mentally unstable and unable to express themselves.

All health problems were turned into problems concerning gender or sexual identity, even if the patient was seeking care for a different, unrelated reason, causing general discomfort with the health experience for the patient concerned:

‘(So you have two organs?) I then explain, I’m male and I want to be female; she turns around and says ‘you just want to change yourself for fun?’ (Nomusa, 24 years, student)

She (the nurse) was asking me, you want breasts, what do you want to do with the breasts?’ (Philile, 23 years, student)

‘I try and explain to him in English, this is an Indian doctor, what I need (help to transition). In front of other patients, mind you! I say, ‘I’m not hermaphrodite, I’m transgender’. He then tells me, tell this to the nurse (what I was saying to him to the nurse, in isiZulu).’ (Philile, 23 years, student)

‘That was the worst day of my life. In this urology unit, nothing is private. No private conversation. This is open space, you [are] surrounded by other patients.’ (Nomfundo, 26 years, unemployed)

‘Yes, I have been to the clinic. It was just for a broken bone, but they made me undress. Maybe they wanted to see if I was really a girl? I don’t know. They were asking me silly questions and coming in and out of the room while I was waiting for the ambulance. Like: ‘uyisitabane?’ (Are you gay?) They don’t understand, they don’t know about us transgender?’ (Mancane, 19 years, scholar)

‘They will then ask, ‘why do you have such a manly name?’ I will tell them, ‘I was born a man’. In most cases that will be the end of the discussion; other[s] will pressure you because they think they can convince you that you are not a woman.’ (Nomusa, 24 years, student)

Participants also reported doubt expressed by the health care worker about the existence of the transgender population’s gender identity, thus being forced to assume the gender identity assigned at birth when seeking medical assistance, as illustrated below in the questions asked by a doctor when the respondent sought help from the health care facility for gender affirmation:

‘They pumped my stomach (following attempted suicide). After recovery, the doctor asked me what was wrong? I was crying and crying. They then called a social worker. I tried to explain my situation. They just did not understand. The doctor said, “is it because you are gay and your family is not accepting you?” I said, “no. I want to be a girl.” The doctor says “no, you are gay. It is okay to be gay”.’ (Nomfundo, 26 years, unemployed)

As described here, the health care environment is set up in a way that recognises only male and female bodies. Participants reported instances of being made to fall in line with their assigned gender in seeking health care.

All participants recounted difficulties with the physical environment of health system facilities, such as appropriate access to bathrooms either as outpatients or as inpatients. Participants resorted to using the bathrooms designated for disabled individuals:

‘I was once admitted to [mentions name] hospital. I requested to be discharged because I couldn’t cope when I needed to bath; I had to wait until the night when there was no one using the bathrooms and bath; I used the female bathrooms. I think at the hospitals they should do like the malls and add family bathrooms. But to be safe I just use the ones for the disabled population. But in the hospital I’ll feel much safer in the ones for females.’ (Mancane, 19 years, scholar)

In these circumstances, the patient’s feelings are denied and minimised, with health care workers assuming a kind of parenting role, effectively bullying the patient and stepping outside professional bounds to proselytise and intimidate, sometimes coming up with religious objections. This has the effect of blocking all lines of communication:

‘But all in all, the old people should just stop trying to save us because they think we are lost, and they can somehow put us back in line according to their beliefs.’ (Sibusiswe, 19 years, student)

‘And we have a problem with the much older nurses; they are the ones who mostly give us problems, they have no respect for us. It’s better the ones who are much younger, they are more informed and they don’t give us problems; they are able to accommodate different sexualities. Older nurses must be made matrons, move them away from the patients, away from us!’ (Nomusa, 24 years, student)

‘He (the doctor) said, God doesn’t approve of this and I was like doctor let’s be neutral now and told him that I’m a transwoman.’ (Philile, 23 years, student)

‘The reason I was there was for medical help. I was not looking for a granny or a mother. I needed medical help!’ (Nobuhle, 23 years, student)

## Discussion

The objective of this study was to examine the experiences of transgender people in accessing health care facilities so that health care workers could be better informed about the needs of transgender patients. It emerged from this study that transgender individuals have unique sexual and reproductive health needs, but difficulty with the health care system. In this study, participants indicated a strong desire to transition; however, because of stigma and lack of appropriate health care some of them end up resorting to particularly dangerous practices, such as using cross-gender hormones without medical supervision, instances of self-mutilation in an attempt to remove genitalia and concealing of sex organs. The findings of this study are similar to what has been reported in other studies that detail that self-mutilation and use of illicitly sourced cross-gender hormone treatments are driven by a lack of access to formal medical health care.^20,21,22^ Participants in this study admitted that they engage in high-risk sexual behaviour such as transactional ‘sex for need’ and unprotected receptive anal and oral sex, thus risking exposure to HIV infection. The practice of unprotected receptive anal and oral sex, linked with a high-risk of HIV infection, has been reported by other studies.^17,18^ The risky sexual behaviours are also linked to societal transphobia and the lack of a social support system, resulting in low self-worth, and thus not able to negotiate safer sex.^17^ In this study, it emerged that transgender individuals have unique sexual partnerships and should be encouraged to disclose sexual practices so that they can be offered targeted health education, including the provision of contraceptives in the case of bisexual transgender females.^28^ Studies have confirmed the finding that the sexual orientation of transgender people may be fluid and should not be linked to the gender assigned at birth nor to gender identity; rather, it must be confirmed with the particular transgender individual before assessing the risk factors and offering relevant safe sex resources and messaging.^28,51^ The findings clearly indicated that there is a need for reproductive health services and effective, risk-free contraceptives, as there is a risk of unplanned pregnancy in transgender men who engage in unprotected receptive vaginal sex with men. In the literature, there is evidence of unplanned pregnancies among transgender men while on exogenous testosterone.^52^

Participants experienced difficulties relating to discriminatory and insensitive treatment by health care providers. They cited discriminatory responses from a range of health professionals, including doctors, nurses, lay counsellors and social workers. The literature confirms that transgender patients have had less than favourable experiences in health care settings where attitudes towards them are driven by stigmatisation and a cisgender-biased health care system.^4,8,9,53,54^ This study revealed that transgender patients were challenged by a health system that lacked basic amenities, such as bathroom access and admission to an appropriate hospital ward. Studies have confirmed that health facilities are commonly established and maintained for a dual gender system of cisgender bodies, which results in failure of the system to accommodate basic sexual and reproductive health care for transgender populations.^9,26^

The participants in this study also reported difficulty in finding health care providers who render competent transgender care and provide a friendly environment. This unfriendly and hostile environment and negative attitudes towards the transgender population have been reported by other transgender researchers.^8,9,10^ It is plausible to suggest that this may be linked to the fact that sexual and gender minorities are not covered in the basic training curriculum for most health care workers, and thus they have not developed professional modesty towards the transgender population.^27,54^ This is also reported by Campbell and Stein, who noted that there are no accredited sexual health courses in South Africa.^55^

Literature indicates that transgender youth experience distress, bullying and isolation, leading to heightened levels of psychological distress, suicide attempts and substance abuse, and possibly also of susceptibility to HIV infection.^56,57^ Similar findings emerged from this study in that the participants were bullied by peers and family members, with one participant reporting attempted suicide. Thus, health workers should be vigilant when caring for young transgender patients and offer psychosocial care.

### Limitations of the study

The study was limited in terms of age diversity, in that all the participants were in their late teens or young adults, with a similar educational background and level of income. The sample also consisted of disproportionately transgender females, and future research should include voices from the transgender male subgroup.

### Recommendations

Development of context-specific transgender care guidelines and policies needs urgent attention. Policies should give guidance on taking sexual history, dosages of cross-gender hormones and related monitoring protocols, including routine screening tests. Sexuality and sexual health should be included as a subject in the South African health care worker curriculum so that future trained professionals will be skilled in the management of the LGBT populations and possibly develop professional courtesy and sensitivity towards sexual and gender minority individuals.^58^ Data routinely collected at health facilities must include gender identity and sexual orientation in order to establish a more accurate database pertaining to the number of transgender and LGBT patients, using health facilities to enable targeted programme planning and funding.

## Conclusion

The transgender population has unique unmet sexual and reproductive health needs. The transgender participants in this study reported experiences of stigmatisation and discrimination in health facilities and noted an absence of skilled health care workers, targeted programmes and resources relating to their needs. The policies and guidelines on STIs, HIV and AIDS must be explicit on the care directed at the transgender population. The guidelines must include contraceptive strategies and routine health checks targeted at the transgender community to minimise harm because of illicit hormone usage, binding of breasts and tucking of male sexual organs. Initiatives to include transgender health in the pre-service training curriculum for health care workers, coupled with in-service training and sensitisation, require urgent attention.
